# No evidence of pulmonary hypertension revealed in an echographic evaluation of right-sided hemodynamics in hyperthyroid cats

**DOI:** 10.1177/1098612X221127102

**Published:** 2022-11-09

**Authors:** Laury Lachance, Bérénice Conversy, Kelly Wiggen, Christophe Pavard, Carol Reinero, Isabelle Masseau

**Affiliations:** 1 Department of Clinical Sciences, Faculty of Veterinary Medicine, University of Montréal, Saint-Hyacinthe, Canada; 2 Department of Veterinary Medicine and Surgery, University of Missouri Veterinary Health Center, College of Veterinary Medicine, University of Missouri, Columbia, MO, USA

**Keywords:** Systolic time intervals, tricuspid regurgitation flow velocity jet, lungs, hyperthyroidism, echocardiography

## Abstract

**Objectives:**

Hyperthyroidism is a common endocrinopathy affecting middle-aged to elderly cats, with multisystemic repercussions. Hyperthyroid humans show decreased lung compliance and increased cardiac output with subsequent left heart failure leading to pulmonary capillary congestion. Prognosis worsens with the development of increased pulmonary vascular pressures (ie, pulmonary hypertension [PH]) in hyperthyroid humans. The effect of excess thyroid hormone concentration on pulmonary arterial hemodynamics is unknown in cats. Assessing pulmonary vascular pressures in veterinary medicine relies heavily on echocardiographic measurements performed at the level of the heart and pulmonary trunk. This study investigated right-sided cardiac and pulmonary arterial hemodynamics in hyperthyroid cats using echocardiography.

**Methods:**

Echocardiographic examinations of hyperthyroid cats identified through a bi-institutional database search were reviewed for the determination of systolic pulmonary arterial pressure (PAP) and 20 other metrics. Values were compared with those of a healthy cat group using non-parametric statistical analyses.

**Results:**

Systolic PAP could not be determined in 23/26 hyperthyroid and 13/14 healthy cats owing to unmeasurable tricuspid regurgitation flow velocity. Hyperthyroid cats were roughly twice as old (*P* <0.001) and had 2–4-fold higher respiratory rates (*P* <0.001) than healthy cats. Hyperthyroid cats showed an increase in acceleration time-to-ejection time ratio of pulmonary flow (1.4-fold, *P* = 0.001), pulmonary artery velocity time integral (1.2–1.6-fold, *P* = 0.001), maximal pulmonary velocity (1.3–1.7-fold, *P* = 0.002), stroke volume (1.5-fold, *P* = 0.001) and cardiac output (1.6-fold, *P* <0.001) vs healthy cats. None of the other echocardiographic metrics reached statistical significance.

**Conclusions and relevance:**

Systolic PAP estimation proved unsuitable as a sole measurement for the assessment of PH in hyperthyroid cats owing to the frequent inability to interrogate tricuspid regurgitant flow velocity. Hyperthyroid cats have altered echocardiographic measures of pulmonary hemodynamics dissimilar to those reported in hyperthyroid humans. Differential effects of thyrotoxic cardiomyopathy on ventricular systolic function may underlie species differences.

## Introduction

Hyperthyroidism is the most common endocrinopathy of middle-aged-to-elderly cats, affecting up to 10% of North American cats.^1,2^ Excess thyroid hormone has multisystemic repercussions shared by hyperthyroid cats and humans, driven by increased metabolism and hemodynamic load.^3,4^ An increased metabolic rate can result in high-output cardiac failure, causing congestion of pulmonary capillaries and pulmonary edema.^4,5^

Pulmonary hypertension (PH) has been reported in 35–65% of hyperthyroid humans.^6–8^ In contrast, the impact of hyperthyroidism on the feline pulmonary circulation is poorly characterized, and, to date, an association with PH has not been reported in cats. Hyperthyroid humans with PH have higher cardiac output (CO), larger cardiac chambers and right ventricular (RV) systolic dysfunction when compared with hyperthyroid patients without PH.^7,8^ While the exact mechanisms leading to PH in hyperthyroid humans remain unclear, cardiac damage and increased pulmonary vascular resistance (PVR) promote a hyperdynamic pulmonary circulation.^6–8^ Indeed, PVR is influenced by increases in catecholaminergic sensitivity of pulmonary capillaries causing pulmonary vasoconstriction,^6,9^ alteration in shear stress forces on the pulmonary vasculature leading to endothelial dysfunction^10,11^ and direct effects of thyroid hormones on pulmonary vascular smooth muscle cells, causing their proliferation.^10,12^

Alterations in pulmonary arterial hemodynamics are reported with several conditions in cats, including congenital cardiac anomalies,^13,14^ parasitism,^15,16^ thromboembolism,^17–19^ inflammatory polyps,^20^ interstitial pulmonary fibrosis^21^ and pulmonary capillary hemangiomatosis.^22^ Included in the assessment of pulmonary arterial hemodynamics is determination of the systolic pulmonary arterial pressure (PAP).

In dogs and cats, PH is defined as systolic PAP >30 mmHg.^23^ Ideally, PAP is measured by catheterization of the RV or pulmonary trunk (PT);^23^ however, in clinical practice, invasive catheterization is rarely performed in these species, leading to reliance on echocardiography as a non-invasive surrogate. In dogs, measurement of tricuspid regurgitation (TR) velocity allows estimation of systolic PAP using the modified Bernoulli equation. Historically, the magnitude of increases in estimated systolic PAP were used to diagnose mild, moderate and severe PH. In a recent consensus statement on PH in dogs, limitations associated with echocardiography were recognized, and reliance on estimated PAP as a sole metric for diagnosis of PH was discouraged.^24^ As cats have the same limitations of echocardiography as dogs and are less likely to have measurable TR jets,^25^ other echocardiographic metrics are all the more important when it comes to assessing pulmonary hemodynamics. Echocardiographic measurements aimed at assessing right-sided cardiac changes complement these. The consensus statement in dogs proposed specific echocardiographic signs related to three anatomic sites (ventricles, PT and right atrium [RA]/caudal vena cava [CVC]) to determine the probability of PH.^24^ These sites allow determination of (1) RV chamber dilation, wall thickening^26,27^ and systolic dysfunction;^27^ (2) PT enlargement,^28^ RV outflow profile type and systolic time intervals (STIs),^28–32^ including pulmonary flow acceleration time (AT; time between onset and peak of pulmonary flow), ejection time (ET; time between onset and end of pulmonary flow) and their ratio (AT:ET; index of the time allocated to acceleration over the total time required for ejection of pulmonary flow); and (3) enlargement of the RA and CVC.^27^ It remains unknown whether criteria for determining the probability of PH in dogs also apply to cats. Application of these other echocardiographic metrics supporting the probability of PH in dogs to cats may ultimately help draft criteria useful to define the probability of feline PH.

The objective of the study was to investigate the potential effects of hyperthyroidism on right-sided cardiac and pulmonary arterial hemodynamics in cats using echocardiography and to explore other echocardiographic metrics that might better define the probability of PH in cats. We hypothesized that hyperthyroid cats would have an elevated estimated systolic PAP and changes in other echocardiographic metrics supporting increased PAP, compared with healthy cats.

## Materials and methods

### Study design

This bi-institutional, retrospective, observational study was conducted at the Centre Hospitalier Universitaire Vétérinaire (CHUV) of the Université de Montréal and the University of Missouri Veterinary Health Center (VHC). Records between 2007 and 2019 were searched for a diagnosis of hyperthyroidism. Newly diagnosed hyperthyroid cats (using the keywords ‘Hyperthyroidism’, ‘Hyperthyroid’ in the official diagnosis; and the filters ‘Species = feline’ and ‘Echocardiographic reevaluation’ or ‘2-D, M-mode echocardiography’) were included if the following criteria were met: (1) complete echocardiogram with an associated report performed at the time of diagnosis (thyroxine [T4] >58 nmol/l); and (2) cats were not receiving treatment for hyperthyroidism at diagnosis. Cats with uncontrolled hyperthyroidism despite receiving antithyroid medication, a comorbidity known to potentially be associated with PH (eg, pulmonary/cardiac parasitism and thromboembolism) or congestive heart failure were excluded. Cats with echocardiographic parameters compatible with a compensated hypertrophic cardiomyopathy (HCM) phenotype such as asymmetric or diffuse left ventricular concentric hypertrophy were included. Demographic data, clinical signs, physical examination findings, T4 concentrations and blood pressure measurements were retrieved from the medical records.

Hyperthyroid cats were compared with healthy cats from a teaching colony (n = 14) recruited in a separate study (St-Arnaud-Massicotte R, Conversy B, Masseau I, et al, unpublished data). The consent form and study protocol were approved by the University of Montreal’s Institutional Animal Care and Use Committee (approval number: 17-Rech-1902). Informed consent was obtained prior to enrollment. Cats were categorized as healthy based upon absence of reported anomalies on clinical history and unremarkable findings on physical examination findings, three-view thoracic radiographs, complete blood count, biochemistry, thyroid hormone levels, fecal testing and heartworm antibody test. Sedation during echocardiography was not allowed for healthy cats, but was allowed in hyperthyroid cats, owing to the retrospective nature of the study.

### Echocardiography

Echocardiographic reports and studies were retrospectively reviewed by an ACVIM-certified specialist in small animal internal medicine experienced in echocardiography (BC, CHUV) or by a third-year veterinary cardiology resident (KW, VHC) for the following information: TR flow velocity (TRFV), AT, ET, AT:ET, maximal pulmonary velocity (MaxPV), pulmonary artery flow profile, RV internal dimension at the end of diastole (RVIDd) and systole (RVIDs), maximal RA diameter, maximal cranial vena cava diameter, pulmonary artery velocity time integral (PAVTI), PT diameter, aortic diameter, interventricular septum (IVS), left ventricular free wall (LVFW) and whether the patient was diagnosed with an HCM phenotype at the time of echocardiography. Echocardiographic images were originally obtained by either a cardiologist, cardiology resident with direct cardiologist supervision or by an ACVIM-certified specialist in small animal internal medicine with experience in echocardiography.

Echocardiographic parameters were recorded as the average of three measurements. Parameters related to the RV, PT and RA were assessed as described in Table 1 to evaluate the probability of PH.^24,33^ Parameters not specific to these anatomic sites were also measured or calculated (Table 2). Systolic PAP was estimated from the modified Bernoulli equation, as indicated in Table 2.

**Table 1:** Acquisition and calculation of echocardiographic parameters to assess right-sided cardiac and pulmonary arterial hemodynamics in hyperthyroid cats by anatomical site

Parameter	Measurement /equation	View
Ventricles^30,33^		
RVIDs	Below the tricuspid valve, in the septal to free-wall direction	Two-dimensional images from a left apical two-chamber cranial view
RVIDd		
RVFS %	(RVIDd–RVIDs)/RVIDd × 100	–
Pulmonary trunk		
AT of pulmonary flow	From onset of pulsed Doppler pulmonary outflow signal to peak flow velocity	Basal right short-axis parasternal view with the PT visible
ET of pulmonary flow	Time interval between onset and end of Doppler pulmonary flow signal	
AT:ET^34,35^	AT:ET	
PAVTI^35^	By tracing the outer border of the flow envelope using the built-in calculation software on ultrasound unit	
MaxPV^35^	Peak velocity of pulmonary outflow from the pulsed or continuous Doppler signal	
AoD^33^	From anterior to posterior wall of aorta at the end of diastole	Right parasternal short-axis view of aorta and LA
PTD^33^	At the beginning of systole	Two-dimensional images of the right parasternal short-axis inflow–outflow view
PTD:AoD ratio	PTD:AoD	–
CSaPT^33^	CSaPT = π(PTD/2)^2^	Right short-axis parasternal view of the LV
RA^33^		
MaxRAD	From the lateral RA wall to the atrial septum, parallel to the tricuspid valve annulus	End-systolic two-dimensional image from a cranial left apical two-chamber imaging view

RVIDs = right ventricle internal dimension at the end of systole; RVIDd = right ventricle internal dimension at the end of diastole; RVFS = right ventricular fractional shortening; AT = acceleration time; PT = pulmonary trunk; AT:ET = acceleration time on ejection time ratio; ET = ejection time; PAVTI = pulmonary artery velocity time integral; MaxPV = maximal pulmonary velocity; AoD = aortic diameter; LA = left atrium; PTD = pulmonary trunk diameter; CSaPT = cross-sectional area of the pulmonary trunk; LV = left ventricle; RA = right atrium; MaxRAD = maximal right atrium diameter

**Table 2:** Acquisition and calculation of additional echocardiographic parameters

Parameter	Measurement/equation	View
Peak TRFV	Peak velocity of the pulsed Doppler signal	Apical four-chamber view or transverse left cranial view
Systolic PAP^24^	(4 × TRFV^2^)	–
PVR^36^	Systolic PAP/PAVTI	–
SV^33^	CSaTP × PAVTI	–
HR	Recorded from ECG leads connected to the ultrasound system	–
CO^37^	SV × HR	–
MaxCrVC^33^	From wall to wall at the entrance of the cranial vena cava into the RA at the beginning of systole (minimum) and end of diastole (maximum)	Left parasternal tilted heart base view
MinCrVC^33^		
CCrVC^33^	(MaxCrVC – MinCrVC)/MaxCrVC × 100	–

TRFV = tricuspid regurgitation flow velocity; PAP = pulmonary arterial pressure; PVR = pulmonary vascular resistance; PAVTI = pulmonary artery velocity time integral; SV = stroke volume; CSaTP = cross-sectional area of the pulmonary trunk; HR = heart rate; ECG = electrocardiography; CO = cardiac output; MaxCrVC = maximal cranial vena cava diameter; MinCrVC = minimum diameter of the cranial vena cava; CCrVC = collapsibility of the cranial vena cava

**Figure 1:**
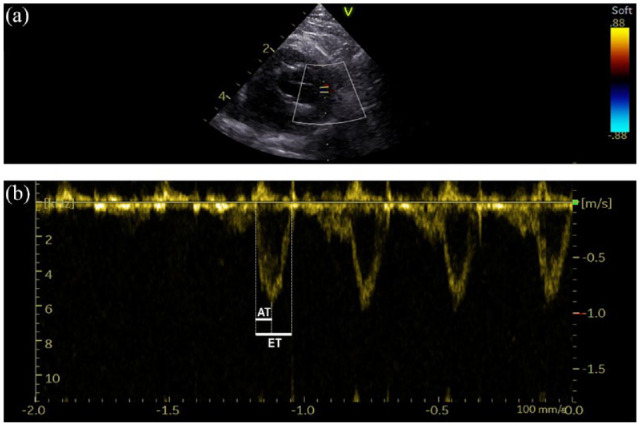
Representative example of an echocardiographic image from a 4-year-old female spayed healthy domestic shorthair cat illustrating the determination of the acceleration (AT) and ejection (ET) time of the pulmonary trunk flow. (a) The placement of the sample volume (short parallel lines) perpendicular to the pulmonary arterial flow on a basal right short-axis parasternal view. (b) The resulting tracing from sample volume placement of the above image. A representative isolated tracing of blood flow ejected into the pulmonary trunk after right ventricle contraction was selected (external dotted lines). The short solid line shows the AT of pulmonary arterial flow, measured from the onset of pulsed Doppler pulmonary outflow signal to peak flow velocity. The ET (long solid line) corresponded to the time between onset and end of Doppler pulmonary flow signal

Pulmonary flow STIs were measured as shown in Figure 1.^34,35^ Pulmonary artery flow profiles were classified as normal (type I) if there was a symmetric envelope with similar acceleration and deceleration time; accelerated (type II) with an asymmetric envelope due to peak velocity early in systole with shortened AT; or as notched (type III), with an asymmetric envelope with rapid acceleration and notching during deceleration.^34,35,38^

IVS and/or LVFW thicknesses ⩾6 mm served for the diagnosis of an HCM phenotype.^39^

### Statistical analysis

Statistical analysis was performed with a statistical software package (SigmaPlot version 14.5; Systat Software). Demographic and echocardiographic numerical variables were compared between hyperthyroid and healthy cats using a Mann–Whitney U-test. Results are reported as median (interquartile range [IQR]), unless stated otherwise. Categorical variables were assessed with Pearson’s χ^2^ test or Fisher’s exact test. After applying a Bonferroni correction for pairwise comparisons, the level of significance was set at *P* <0.004 and *P* <0.0022 (0.05/n) for demographic and physical examination findings (n = 12) and echocardiographic metrics (n = 22), respectively. The relationship between thyroid hormone concentrations and echocardiography parameters and between the age of cats and STIs was assessed with a Spearman and Pearson correlation, respectively. Grubbs’s or Rout’s tests were used to assess for outliers. Multiple linear regression models were used to study the relationships between systolic time intervals (AT, ET, AT:ET) and the independent variables (stroke volume and MaxPV).

## Results

Fourteen hyperthyroid cats from CHUV and 12 from VHC were compared to 14 healthy cats (Table 3). Circulating T4 concentrations ranged from 58.9 to 555.0 nmol/l (median 73.4) in hyperthyroid cats and from 18.5 to 48.4 nmol/l (median 28.5) in healthy cats. Sedation with butorphanol (0.2–0.3 mg/kg IV) was required in three hyperthyroid cats.

**Table 3:** Demographics and relevant physical examination findings of healthy and hyperthyroid cat groups

Variable	Healthy (n = 14)	Hyperthyroid (n = 26)	*P* value
Demographics			
Age (years)	5.3 (3.6–7.1)	13 (10–14)	<0.001
Sex (female/male)	10/4	12/14	0.59
Purebred*	1 (7)	6 (23)	0.23
Body weight (kg)	3.8 (3.6–4.8)	4.2 (3.4–4.7)	0.91
Neutered	14 (100)	26 (100)	>0.99
Physical examination findings			
Rectal temperature (°C)	38.5 (38.3–39.2)	38.2 (37.6–38.7)	0.218
HR (bpm)	200 (193–209)	190 (176–218)	0.45
Respiratory rate (rpm)	16 (14–18)	46 (30–70)	<0.001
Heart murmurs	0 (0)	18 (69)	–
Thyroid nodules	0 (0)	15 (58)	–
BCS^†^	5 (5–5)	3.5 (2–5)	0.10
Systolic systemic arterial BP (mmHg)	150.5 (142.0–165.2)	148.4 (130.0–187)	0.917

Data are presented as n (%) or median (interquartile range)

* In the hyperthyroid group, purebred cats were represented by one subject for each of the following breeds: Siamese, Norwegian Forest Cat, Maine Coon, Persian, Himalayan and Tonkinese; the healthy group included one Burmese cat

† Body condition score (BCS) was assessed on a standard scale of 9^40^

HR = heart rate; bpm = beats/min; rpm = resps/min; BP = blood pressure

Clinical signs compatible with hyperthyroidism, including weight loss with ravenous appetite (n = 10), vomiting (n = 7), emaciation (n = 4), dull haircoat (n = 4), vocalizing (n = 3) and behavior changes (n = 2), were observed in 20/26 (77%) hyperthyroid cats. Respiratory signs were noted in 8/26 (31%), including increased bronchovesicular sounds (n = 5), labored and/or open-mouth breathing (n = 4), inspiratory noises (n = 1), cough (n = 1), sneezing (n = 1) and exercise intolerance (n = 1).

**Figure 2:**
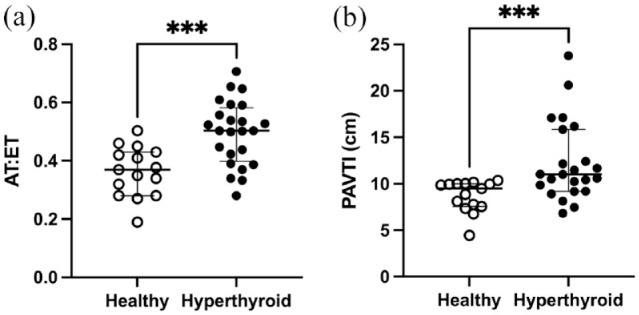
Distributions of (a) ratio of acceleration time (AT) to ejection time (ET) of pulmonary flow (AT:ET) ratio and (b) pulmonary artery velocity time integral (PAVTI) in healthy and hyperthyroid cats. Parameters were assessed in all healthy cats (n = 14). AT:ET and PAVTI were measured in 24/26 and 23/26 hyperthyroid cats, respectively. Horizontal lines and error bars represent median and interquartile range. These parameters were significantly higher in hyperthyroid cats than in healthy cats

Among the echocardiographic parameters presented in Table 4, AT:ET, PAVTI, MaxPV, SV and CO were, on average, 1.2–1.7-fold higher in hyperthyroid cats vs healthy cats after applying the Bonferroni correction for multiple individual pairwise comparisons. Associations between these variables (ie, AT:ET, PAVTI, MaxPV, SV and CO) and thyroid hormone concentration values yielded Spearman correlation coefficients of 0.53 (*P* = 0.001), 0.55 (*P* <0.001), 0.36 (*P* = 0.03), 0.48 (*P* = 0.006) and 0.51 (*P* = 0.003), respectively. Hyperthyroid cats had an approximately 1.4-fold increase in AT:ET vs healthy cats with values ranging from 0.19 to 0.50 in healthy cats and 0.28 to 0.71 in hyperthyroid cats (Figure 2a). Multiple regression analysis models were weak for AT, ET and AT:ET with an R^2^ of 0.20, 0.27 and 0.20, respectively, with regard to their relation to SV and MaxPV. Both SV and MaxPV were determinants of ET (*P* = 0.01 and *P* = 0.003, respectively) with coefficients of −29.53 and 4.99, respectively. Regarding SV and MaxPV, only the latter was a determinant of AT:ET (*P* = 0.02) with a coefficient of 0.13. Neither SV or MaxPV was a determinant of AT.

**Table 4:** Echocardiographic data of hyperthyroid and healthy cats

Parameters	Healthy (n = 14)	No of cats assessed (%)	Hyperthyroid (n = 26)	No of cats assessed (%)	*P* value
RV data					
RVIDs (mm)	4.76 (3.96–5.79)	14 (100)	3.48 (2.78–4.53)	18 (69)	0.006
RVIDd (mm)	7.28 (6.82–8.74)	14 (100)	7.05 (6.48–7.64)	18 (69)	0.210
RVFS (%)	38.69 (31.07–44.14)	14 (100)	47.63 (37.74–57.52)	18 (69)	0.015
PT data					
AT (ms)	51.74 (41.46–67.13)	14 (100)	66.54 (58.04–80.07)	24 (92)	0.007
ET (ms)	142.53 (135.40–160.36)	14 (100)	138.34 (119.08–166.67)	24 (92)	0.283
AT:ET	0.37 (0.28–0.44)	14 (100)	0.50 (0.40–0.58)	24 (92)	0.001
PAVTI (cm)	9.18 (7.50–9.98)	14 (100)	11.01 (9.21–15.84)	23 (88)	0.001
MaxPV (m/s)	0.85 (0.68–1.03)	14 (100)	1.17 (0.89–1.72)	25 (96)	0.002
AoD (mm)	9.07 (8.52–9.80)	14 (100)	9.48 (9.03–10.13)	26 (100)	0.251
PTD (mm)	7.93 (7.30–8.90)	14 (100)	8.35 (7.19–9.09)	22 (85)	0.673
PTD:AoD ratio	0.91 (0.80–0.98)	14 (100)	0.87 (0.80–0.98)	22 (85)	0.697
CSaPT (cm^2^ )	0.49 (0.42–0.62)	14 (100)	0.55 (0.41–0.65)	22 (85)	0.673
RA data					
MaxRAD (mm)	13.82 (11.59–14.38)	14 (100)	11.73 (11.11–12.57)	20 (77)	0.086
Other parameters					
TRFV (ms)	1.73	1 (7)	1.80 (1.70–2.20)	3 (12)	NA
Systolic PAP (mmHg)	12.44	1 (7)	13.03 (11.56–19.70)	3 (12)	NA
PVR (WU)	0.42 (0.37–0.68)	11 (79)	1.37 (0.38–2.35)	4 (15)	0.55
SV (ml)	4.26 (3.60–4.77)	14 (100)	5.99 (5.31–6.93)	19 (73)	0.001
HR (bpm)	192 (168–210)	14 (100)	206 (164–230)	26 (100)	0.178
CO (l/min)	0.76 (0.62–0.91)	14 (100)	1.13 (0.96–1.48)	19 (73)	<0.001
MaxCrVC (mm)	6.38 (4.95–6.93)	14 (100)	5.92 (4.33–6.57)	7 (27)	0.456
MinCrVC (mm)	3.63 (3.38–4.27)	14 (100)	3.68 (2.86–4.77)	7 (27)	0.709
CCrVC (%)	37.89 (32.37–41.94)	14 (100)	36.59 (30.68–38.55)	7 (27)	0.296

Data are presented as median (interquartile range)

RV = right ventricle; RVIDs, right ventricular internal dimension at the end of systole; RVIDd, right ventricular internal dimension at the end of diastole; RVFS, right ventricular fractional shortening; PT = pulmonary trunk; AT = acceleration time; ET = ejection time; AT:ET = acceleration time on ejection time ratio; PAVTI = pulmonary artery velocity time integral; MaxPV, maximum pulmonary velocity; AoD = aortic diameter; PTD = pulmonary trunk diameter; CSaPT = cross sectional area of the pulmonary trunk; RA = right atrium; MaxRAD = maximal right atrial diameter; TRFV = tricuspid regurgitation flow velocity; NA = not applicable; PAP = pulmonary arterial pressure; PVR = pulmonary vascular resistance; WU = Wood units; SV = stroke volume; HR = heart rate; bpm = beats/min; CO = cardiac output; MaxCrCV = maximal cranial vena cava; MinCrCV = minimal cranial vena cava; CCrVC = collapsibility of the cranial vena cava

Hyperthyroid cats had a 1.2–1.6-fold higher PAVTI than healthy cats, with values not following a normal distribution based on the Shapiro–Wilk test (*P* <0.05). Hyperthyroid cats showed a wider range of values (7.47–23.78) than healthy cats (4.44–10.16) without identification of outliers based upon Rout’s test (Figure 2b). Interestingly, one healthy cat had an RVFS of 66%, which was considered to be an outlier based on Grubb’s test, while all others of the same group had values <50% (normal reference interval [RI] 45–50%).^39,41^ In comparison, 7/18 hyperthyroid cats had an RVFS of >50%. Four of these seven cats had RVIDs and RVIDd values lower than the lowest IQR value of the healthy cat group, while this was the case only for RVIDs in the remaining three cats. Pulmonary flow profiles did not differ between groups and were type I and II for 24/26 and 2/26 hyperthyroid cats, respectively, and type I for 14/14 healthy cats. Of note, MaxPV ranged from 0.7 to 2.5 ms in hyperthyroid cats (17/26 individuals >1 ms and 9/17 individuals >1.5 ms) and from 0.5 to 1.3 ms in healthy cats (3/14 individuals between 1 and 1.3 ms).

TRFV was measurable in only 3/26 (12%) hyperthyroid and 1/14 (7%) healthy cats. In the former group, it did not exceed 2.2 ms (representing an RA to RV pressure gradient of 19.7 mmHg). Thirteen healthy cats had TR jets that were too small to be adequately measured. The age of the healthy cats was not correlated with STIs. An HCM phenotype was diagnosed based on a LV wall thickness of ⩾6.0 mm in 17/26 (65%) hyperthyroid cats and none of the healthy cats.

## Discussion

Among echocardiographic metrics assessed related to right-sided cardiac and pulmonary arterial hemodynamics, our study revealed a significant increase in AT:ET, PAVTI, SV and CO in hyperthyroid cats compared with healthy cats. The absence of or inability to measure TRFV jet in most cats prevented estimation of systolic PAP, hampering our capacity to test our first hypothesis. Given the suspicion that mitral regurgitation with HCM may be attributed to a secondary alteration in the mitral valve annulus,^42^ it is reasonable to predict that, if hyperthyroid cats experienced RV changes secondary to altered pulmonary arterial hemodynamics, TR might be more common and more easily interrogated in hyperthyroid cats. However, this is the opposite of what we noted in this study, with only a few hyperthyroid cats having a measurable TR jet velocity. Potential reasons for this finding could include challenges in consistent alignment with flow (anecdotally, the authors feel that cats with hyperthyroidism have more cardiac movement, precluding reliable interrogation of small or intermittent regurgitant jets), and challenges with patient temperament in hyperthyroid cats.

An additional parameter used in the diagnosis of PH in dogs is the shape of the pulmonary artery flow profile; a type I profile is associated with normal PAPs, whereas type II and III profiles are associated with progressively higher levels of PH.^24^ While this parameter has not been evaluated in cats, the authors collected these data to be as complete as possible; no significant difference was noted between healthy and hyperthyroid cats, supporting the lack of overt evidence of PH in cats with hyperthyroidism. With regard to our second hypothesis, changes found in our study are dissimilar to those reported in humans with elevated systolic PAP or in dogs with a moderate or high probability of PH.

Interestingly, several hyperthyroid cats had an RVFS superior to normal RI, which is in contrast to hyperthyroid humans with PH who demonstrate RV systolic dysfunction, leading to worsening RVFS.^8^ In humans, this phenomenon appears to occur following initial myocardial hypertrophy, followed by progressive impairment of RV contractile function and subsequent dilatation.^43^ Dilation of the RV was not observed in the hyperthyroid group. Mathematically, an increase in RVFS can result from several scenarios, including a decrease in RVIDs only, an increase in RVIDd only, an increase in both parameters with the preferential increase of RVIDd over RVIDs, or a decrease in both RVIDs and RVIDd, with a more profound decrease in RVIDs than RVIDd. The last scenario applies to 5/7 hyperthyroid cats with elevated RVFS, while the remaining two had a decrease in RVIDs only. This result may represent a consequence of an exaggerated recruitment of the cardiac muscle, first with increasing basal metabolic rate and, second, following a direct effect of thyroid hormones on myocardium (ie, positive inotropic and chronotropic effects, stimulation of myocardial hypertrophy and an increased response to adrenergic stimulation).^44–47^ Increasing energy demand associated with elevated T4 concentrations reportedly drives the development of thyrotoxic cardiomyopathy.^41,44,46,48^ Thyrotoxic cardiomyopathy can affect up to 45% of hyperthyroid cats, especially with severe hyperthyroidism, and echocardiographic changes are reversible following return to an euthyroid state.^49^ In the event that systolic PAP remains within the normal RI, an increase in RVFS may reflect a potential decrease in afterload where blood is more easily ejected from the RV into the PT.

Among the PT parameters, a significant increase in the AT:ET ratio was unexpectedly observed in hyperthyroid cats vs healthy cats. This suggests that a greater proportion of time allotted for blood flowing from the RV into the PT is required to reach MaxPV. This is in contrast to dogs and humans with PH in which AT and the AT:ET ratio are negatively correlated with increases in systolic PAP.^31,32,35,38^ In these species, short STIs result from increased PVR and reduced distensibility of the pulmonary vascular bed, provoking an elevated RV afterload and thus an earlier reach of MaxPV.^30,38,50^ In accordance with a prolonged time to reach MaxPV, over half (n = 17/26) of the hyperthyroid cats had a MaxPV above the normal reference value (>1 ms),^41^ including nine cats in which the value was >1.5 ms. No study has reported similar changes in dogs or humans with PH. It is plausible that hyperthyroidism causes an increase RV contractility contributing to an increased pulmonary arterial flow velocity in the absence of increased PVR in these cats. Additionally, it is also possible that hyperthyroid cats may have been more prone to developing dynamic RV outflow tract obstruction (DRVOTO) as a consequence of a hyperdynamic state induced by the hyperthyroidism.^51^ The phenomenon of DRVOTO is considered a benign cause of murmurs in cats, and exhibits a Doppler profile that is dynamic, with late-peaking profiles at faster HR secondary to progressive narrowing of the RV over the course of systole.^51^ A late peaking profile would result in increased AT:ET, and may explain the findings of this study. Future studies are needed to determine the frequency of DRVOTO in hyperthyroid cats, and what type of alterations in pulmonary flow profiles may occur if DRVOTO and PH occur in the same patient.

The higher SV in hyperthyroid cats vs healthy cats was expected. With hyperthyroidism, elevation of the basal metabolic rate leads to an increase in blood volume due to renin–angiotensin–aldosterone system activation.^5,44,48^ An increase in SV without significant variation in HR leads to increased CO. Hyperthyroid humans with PH show a similar increase in CO, although this is related to hyperthyroidism-induced tachycardia rather than an increased SV, as found in our study.^8,52^

Although this study focused on echocardiographic parameters related to pulmonary flow alterations, it is important to consider that the pulmonary circulation, especially PAP, can also be influenced by PVR and pulmonary venous pressure.^53^ These parameters are not routinely directly measured in veterinary practice as they require invasive cardiac catheterization. In our study, the increase in pulmonary flow in hyperthyroid cats secondary to elevated SV would favor the development of increased systolic PAP. The absence of measurable systolic PAP in most hyperthyroid cats, and the fact that the determination of PVR is based on integration of the systolic PAP, limits our ability to draw conclusions. Larger numbers of hyperthyroid cats in which PVR could be calculated (or measured invasively) would be necessary to better assess the role of PVR in observed changes in pulmonary flow STIs.

The study had several limitations. First, hyperthyroid cats were older than healthy cats. While age-matched cats would have been ideal, recruitment of healthy cats aged >10 years without any comorbid conditions would be challenging. Healthy cats included in the study ranged in age from 3 to 11 years old, with only one cat greater than 10 years of age. To our knowledge, no data have been reported to date regarding the influence of age on systolic PAP in cats. In humans, age-related increases in PVR and systolic PAP are observed in the elderly. Increased stiffness of pulmonary vessels secondary to pulmonary remodeling and a decreased CO caused by changes in systemic circulation and left ventricular diastolic dysfunction have been reported.^54–56^ A confounding effect of age and hyperthyroidism on measured parameters is possible. Second, as the study was retrospective, echocardiographic examinations from the hyperthyroid group were not optimized to specifically evaluate indices of right-sided and pulmonary arterial hemodynamics; additionally, many hemodynamic parameters that have been evaluated in dogs for the diagnosis of PH such as the velocity of pulmonic valvular insufficiency, pulmonary artery flow profiles and right pulmonary artery distensibility index have not been evaluated in cats. Several parameters could not be assessed in all patients due to inadequate echocardiographic image quality. Third, all potential comorbidities could not be entirely excluded in several hyperthyroid cats owing to a lack of fecal examination and/or heartworm testing. Fourth, investigators reviewing echocardiographic examinations and collecting measurements were aware of the study aim and health status of the patient (information bias). Finally, echocardiography itself is a limitation as it only serves to estimate certain pulmonary arterial hemodynamic parameters and may vary from measurements obtained during invasive catheterization; although ideal, right heart catheterization^32^ is ethically debatable and unrealistic in client-owned cats.

## Conclusions

This study revealed an increase in the AT:ET ratio and PAVTI in cats with hyperthyroidism, in contrast to what is seen in hyperthyroid humans. These findings support the existence of interspecies differences in response to hyperthyroidism and may be driven at least in part by SV-derived increased CO. Assessment of systolic PAP was limited by the inability to determine peak TRFV in the majority of hyperthyroid cats.
